# Effects of different levels of egg protein replacement in weaned diets on hematology, kidney functions, and immunity biomarkers

**DOI:** 10.1002/fsn3.3204

**Published:** 2023-01-06

**Authors:** Muhammad Abdul Rahim, Muhammad Naeem, Khunsha Khalid, Muhammad Imran, Muhammad Kamran Khan, Muhammad Imran Khan, Mahr Un Nisa, Muhammad Sarwar, Chinaza Godswill Awuchi

**Affiliations:** ^1^ Department of Food Science, Faculty of Life Sciences Government College University Faisalabad Pakistan; ^2^ National Institute of Food Science & Technology University of Agriculture Faisalabad Pakistan; ^3^ University Medical and Dental College Faisalabad Pakistan; ^4^ Department of Mathematics & Statistics, Faculty of Sciences University of Agriculture Faisalabad Pakistan; ^5^ Department of Nutritional Sciences, Faculty of Medical Sciences Government College University Faisalabad Pakistan; ^6^ Postgraduate Studies and Research The University of Lahore Lahore Pakistan; ^7^ School of Natural and Applied Sciences Kampala International University Kampala Uganda

**Keywords:** blood parameters, completely randomized design, immunity biomarkers, kidney functions, nonisonitrogenous and isocaloric diets

## Abstract

Eggs are good sources of nutrients essential for the growth and development of infants. Introducing eggs as a weaning food can improve dietary adequacy in infants at risk for protein energy malnutrition (PEM). To evaluate the current objective, 72 pups (36 males and 36 females) were used to calculate the impact of various egg protein levels on blood parameters. Nonisonitrogenous and isocaloric pellet diets were offered to pups for 28 days using nine pups with three replicates according to a completely randomized design (CRD). The water intake and ad libitum diet were offered to weaned pups. The pups were randomly assigned to different concentrations of diet, which contained WF_0_, control diet, 14% of soybean protein; WF_1_, 14% of egg protein; WF_2_, 16% of egg protein; and WF_3_, 18% of egg protein, respectively. After weaning, the intraperitoneal injection with the drug (xylazine with ketamine) was used to anesthetize before killing on the 28th day. Blood samples were used to measure the blood metabolites. The results indicated that the concentration of red blood cells, white blood cells, serum triglycerides, and serum protein was significantly (*p* ≤ .05) increased in pups fed with high egg protein levels compared to the control. The highest platelet count was observed in the pups fed WF_3_ diet. In contrast, the amount of alanine aminotransferase and aspartate aminotransferase was significantly (*p* ≤ .05) reduced with increasing the level of egg protein in the diets of weaned pups. Immunity biomarkers (immunoglobulin A, immunoglobulin G, and immunoglobulin M) and kidney functions (creatinine and blood urine nitrogen) were nonsignificantly (*p* ≤ .05) increased in the pups fed a high level of protein due to a high biological value of soybean protein. Moreover, the concentration of immunoglobulin E in all pups remained unchanged. Egg protein in infant formula feed can be used for the growth and development of infants.

## Graphical abstract

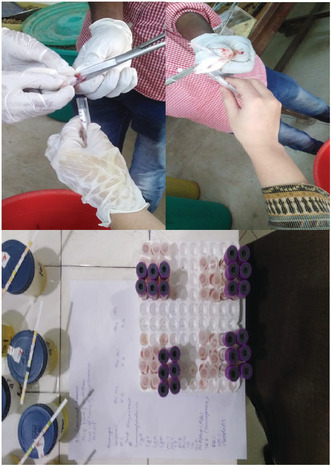

## 1 INTRODUCTION

In recent decade, protein energy malnutrition (PEM) is identified as a major health and nutritional problem in developing countries because of factors such as the lack of availability of adequate breast milk substitutes, microbial contamination of foods and fluids, and displacement of breast milk by less nutritious alternatives, and its rate is increasing. Therefore, 6 million young infants die each year due to inadequate and unbalanced dietary intake of nutrients, and that mortality is mostly observed during the weaning period with an index of seriousness (González‐Torres et al., 2014; Semba, 2016; Tomkins, 2000).

During the transit face, most mothers are transferred their baby's feed from nutritious breast milk to weaning formula diets. Infants need a balanced diet for growth and development, especially in the first 2 years of life. Most of the weaning formula diets available in the market is not contained a balanced protein and energy. Most of the weaning food contains cereals, rice, and fruits that are major ingredients found in the weaning formula feed, which are deficient in particular protein contents and amino acids such as tryptophan and lysine. Balanced formula feed at transit face from mother feed to weaning food is only possible if the nutritionist considers the protein sources in the diet of weaning food. Soybean and other pulses have been recognized as good plant protein sources that can fulfill the protein requirement at this particular weaning stage of infants (Awuchi & Okpala, 2022; Vandenplas et al., 2021). For the growth, activity, and reduced risk of PEM, cereals and soybean in baby formula feed are being used in many intervention programs to improve the nutritional status of infants and reduce the risk of poor growth (Awuchi, 2022). Soybean has been recognized as a rich source of essential amino acids such as tryptophan and lysine, but it is not grown conventionally in Pakistan (Ajmal et al., 2022; Dutta & Sharma, 2020; Ghosh‐Jerath et al., 2017; Muhimbula et al., 2011; World Health Organization, 2012). Therefore, these essential amino acids are lacking in baby formula diets in Pakistan. There is a need to move toward nutritious alternatives source to fulfill the nutritional requirements of infants for growth and development. Among alternative sources, eggs have excellent bioavailability of essential amino acids and are good for an infant's health, according to the World Health Organization (WHO), and can be mixed with weaning food. Currently, Pakistan is producing 4472 million eggs domestically and 16,797 million eggs commercially, but unfortunately the country's per capita consumption is only 65–70 eggs annually against the global average consumption of around 160 eggs. Therefore, egg protein can be introduced in weaning food to reduce the risk of PEM by having a significant role in the infant growth, repair, and maintenance of body tissue (GPFD, 2021; Headey et al., 2018). In our recent research, non isonitrogenous and isocaloric diets were prepared using extruded egg powder as an animal source (Naeem et al., 2021). In this study, different levels of protein in pup's diets replaced with soybean protein from egg sources were used to see the effects on blood metabolites, liver safety enzymes, kidney functions, serum triglycerides, serum protein, and immune biomarkers of pups models.

## 2 MATERIALS AND METHODS

### 2.1 Animals, foods, and design

Wistar albino pups were purchased from the National Institute of Health, Islamabad, Punjab, Pakistan. Pups were placed under stable or constant conditions, relative humidity was 45–55%, and the temperature was 25 ± 1°C, with a 12‐h/12‐h light–dark cycle (lights on at 06:00 pm). All the experimental protocols were approved by the Ethical Committee of the Government College University, Faisalabad, Punjab, Pakistan, in accordance with the guidelines of the Principles of Laboratory Animal Care (NIH). A total of 72 Wistar albino pups (initial body weight = 25 ± 10 g) at the age of 18 ± 1 days were used in this research. Male and female pups were housed in a white plastic cage for weaning. Weaned pups were offered food and water with standard ad libitum for 28 days to evaluate the impact of high egg protein levels on various parameters. Claassen (1994) reported that the standard feed intake is 5 g/100 g by body weight, while the standard water intake is 10 ml/100 g by weight.

According to the research work of Naeem et al. (2021), nonisonitrogenous and isocaloric pellet diets were formulated to meet the nutrient requirements of the National Research Council (1995). Pups were distributed randomly according to the complete randomized design (CRD) into the four experimental groups consisting of 18 pups each (male, *n* = 9 and female, *n* = 9) and fed experimental diets until 28 days. Four groups were defined: WF_0_, control diet, 14% of soybean protein; WF_1_, 14% of egg protein; WF_2_, 16% of egg protein; and WF_3_, 18% of egg protein (Table 1). At the end of the study, the pups were anesthetized by intraperitoneal (IP) injection of a drug combining xylazine with ketamine before killing on the 28th day of weaning. Throughout the research work, the pups were monitored daily for food intake, and body weight was calculated twice a week.

**TABLE 1:** Ingredients quantities (W/W) of formulated weaned foods and their composition for pups

Ingredients	WF_0_	WF_1_	WF_2_	WF_3_
Rice (C.P 8%)	50	50	50	50
Carrot (C.P 6%)	4.5	4.5	4.5	4.5
Egg powder	0	17	22.35	34
Soybean meal (C.P 44%)	34	17	11.65	0
Dextrose monohydrate	10	10	10	10
Xanthan gum	1.5	1.5	1.5	1.5
Chemical composition				
Ash contents	2.66	1.92	1.92	2.17
Fat contents	1.31	2.79	2.79	3.54
Moisture contents	8.41	8.09	8.08	8.51
Protein contents	14.16	14.95	16.76	18.67
Dietary fiber	5.31	4.12	4.12	4.18

*Note*: WF_0_, control diet, 14% of soybean protein, WF_1_, 14% of egg protein; WF_2_, 16% of egg protein; and WF_3_, 18% of egg protein.

### 2.2 Nutrient composition of food

A nutrient composition, such as protein, fiber, moisture, ash, and fat contents of formulated food, was assessed using the standard method of AOAC (2006) and Awuchi et al. (2020) with minor modifications as described in Table 1 (Naeem et al., 2021).

### 2.3 Blood sample collections

**FIGURE 1:**
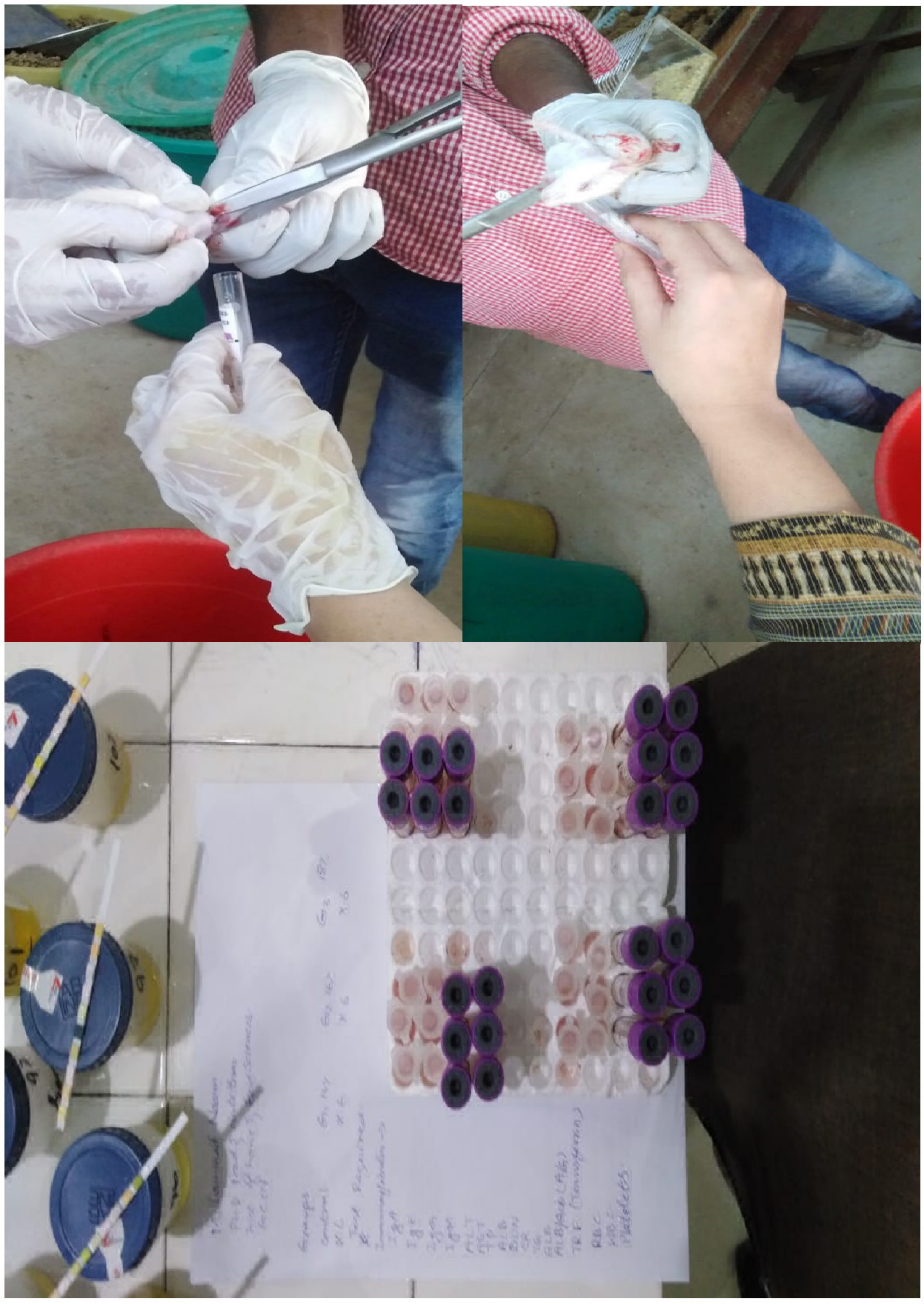
Collection of blood samples from pups’ jugular vein

At the end of the study, the blood samples were collected from jugular vein of pups. The jugular vein was punctured with a 25G syringe (made by Becton Dickinson, Punjab, Lahore, Pakistan), and blood was drawn in vessels slowly to prevent the collapse of the vein and placed in ethylenediaminetetraacetate (EDTA) (Figure 1). Blood samples were collected for serum protein and enzyme determination and centrifuged for 30 min. Collected serum samples were frozen at −20 for further analysis (Uchida et al., 2001).

### 2.4 Biochemical analysis of blood

The enzyme‐linked immunosorbent assay (ELISA) was used to determine the serum immunoglobulins, such as immunoglobulin A (IgA), immunoglobulin G (IgG), and immunoglobulin M (IgM). According to the research work of Cuevas et al. (2013), a standard method was used for the analysis of white and red blood cells. Furthermore, platelet count was determined by following the protocol of Goncalves et al. (2011). The levels of aspartate aminotransferase (AST) and alanine aminotransferase (ALT), total protein, albumin, blood urea nitrogen (BUN), creatinine, triglyceride, globulins GLB, ALB/GLB and A/G, and transferrin in serum were detected using an automatic biochemistry analyzer (Hitachi, Japan).

### 2.5 Statistical analysis

Red blood cells (RBCs), white blood cells (WBCs), platelets, ALT, AST, creatinine, BUN, triglycerides, total protein, albumin, globulin, albumin/globulin, transferrin, IgA, IgG, and IgM were analyzed by the analysis of variance (ANOVA) of linear model with a completely randomized design (CRD) using the Statistics Version 8.1. All data were expressed as mean ± SD, as presented in Steel et al. (1997). Differences were considered significant (*p* ≤ .05) using Tukey's mean comparisons.

## 3 RESULTS AND DISCUSSION

### 3.1 Biochemical analysis

#### 3.1.1 Blood parameters

The impact of high egg protein diets on the blood parameters of pups has been described in Table 2. There were significant (*p* ≤ .05) changes observed in the amount of red blood cells (RBCs) in weaning pups. The highest amount of RBCs in pups (7.35 ± 0.08 million/mm^3^) was observed in the WF_3_ diet, while the lowest value of RBCs was 6.52 ± 0.06 million/mm^3^ in pups fed with a 100% plant protein diet. Furthermore, an increasing similar trend of white blood cells (WBCs) was observed in pups fed egg protein‐containing diets compared to the control diet. On the addition of 28 days, animal and plant protein showed a positive impact of high egg protein diet on the growth and development of pups. However, minimal changes in platelet count or variables were observed during the 28‐day pretrial periods. The concentration of platelet count was significantly (*p* ≤ .05) increased in the high level of egg protein diet by 18% from 943.01 ± 1.22 × 10^9^/L to 948.22 ± 1.92 × 10^9^/L, respectively. Blood parameters such as RBCs, WBCs, and platelet count have been investigated as potential risk markers in several clinical conditions. RBCs play a crucial role in gas transportation as well as nutrients throughout the body of an individual due to their unique shape and structure (Barbalato & Pillarisetty, 2020). The diets having different components of egg such as egg white, ovalbumin, and egg yolk with the same nitrogen contents showed a nonsignificant (*p* ≤ .05) effect on RBCs at the end of 42‐day trial of rats (Kobayashi et al., 2015). Contrary results were found by Ufele et al. (2008), and RBCs in mice with different levels of plant protein diets (soybean and corn meal) were higher and significantly (*p* ≤ .05) different. In another research, children fed animal protein‐based lunch had higher RBCs than those fed the vegetable protein‐based lunch. Higher RBCs may be due to higher bioavailability of animal protein than vegetable protein (Aguirre et al., 1959). It has been well documented that animal protein sources contain more iron than vegetable protein. Kim et al. (2015) and Song et al. (2014) also found the same results in WBCs count when they used different concentrations of egg white (0, 10, 50, and 100 mg/d) in diets of weaned pups. However, the number of B‐lymphocytes increased significantly (*p* ≤ .05) compared to the control diet at the end of the fourth week. Furthermore, results have been documented by Kim et al. (2014) that a lower count of WBCs was observed in the high level of plant protein diet.

**TABLE 2:** Effect of high egg protein on blood cells in pups

Treatments	Hematological parameters		
	RBCs (million/mm^3^)	WBCs (million/mm^3^)	Platelets (10^9^/L)
WF_0_	6.52 ± 0.06^d^	9.32 ± 0.14^a^	943.01 ± 1.22^c^
WF_1_	6.71 ± 0.10^c^	10.42 ± 0.16^d^	945.78 ± 1.71^b^
WF_2_	7.03 ± 0.01^b^	13.78 ± 0.17^c^	947.33 ± 1.94^ab^
WF_3_	7.35 ± 0.08^a^	14.30 ± 0.25^b^	948.22 ± 1.92^a^

*Note*: WF_0_, control diet, 14% of soybean protein; WF_1_, 14% of egg protein; WF_2_, 16% of egg protein; and WF_3_, 18% of egg protein. ^A to D^Means with different superscripts differ significant effect (*p* ≤ .05).

Abbreviations: RBCs, Red blood cells; WBCs, White blood cells.

Red blood cells are recognized as the most important component in the blood and the vertebrate's principal means of transporting oxygen and nutrients to the body's tissues in exchange for carbon dioxide back to the lungs. Changes in the amount of RBCs can be caused by thickening of the blood, vitamin deficiency anemia, slow flow of blood, and eventually blood clots (Hoffman et al., 2013). In addition, WBCs count is known for their participation in immune system processes, including both innate as well as humoral immunity. They circulate in the blood and are also known as leukocytes. WBCs are a vital portion of the body's immune system response. They work against infection and protect the body from other foreign substances. The WBC should be normal, if the number of WBCs is low or high, it can lead to various health problems such as infections, inflammation, chronic cancer, pain, fever, and difficult to breath (Ashton, 2007; Tigner et al., 2021). Moreover, platelets or small cells in the body also play a crucial role to maintain the volume of blood through the activation of blood clotting cascade in any type of vascular injury (Fountain & Lappin, 2020). In conclusion, it was found that our research work on whole‐egg protein with 18% level might perform best in blood parameters.

#### 3.1.2 Liver safety enzymes

The impact of increasing the egg protein level on the liver function of pups is shown in Table 3. In this study, alanine aminotransferase (ALT) was significantly (*p* ≤ 0.05) reduced in pups fed the high level of egg protein than in those fed the plant protein diet. The lowest ALT was 32.11 ± 0.78 IU/L in pups fed the WF_3_ diet, and the highest was 124.22 ± 1.56 IU/L in pups fed the WF_0_ diet. A similar trend of aspartate aminotransferase (AST) was observed in pups fed a diet containing 18% egg protein compared to the control diet. The results showed that the protein source and its level significantly (*p* ≤ 0.05) altered the liver safety enzymes in pups. Our results well agreed with the research of MacQueen et al. (2011), a diet consisting of vitamins, minerals, carrots, and eggs showed that the activity of AST and ALT in weaning rats was significantly reduced with increasing the level of eggs. In another similar research work, the concentration of ALT and AST was nonsignificantly affected by the main supplements of protein (milk and egg products), with and without soy protein diet. The amount of ALT and AST found in the diet of pups was in the acceptable range (Pastuszewska et al., 2008). Moreover, the concentrations of ALT and AST were nonsignificantly different in weaned rats fed with egg white with and without *bacillus thuringiensis* Cry1Ia12 entomotoxin protein (Guimaraes et al., 2010). All diets used in this research work either contain animal or plant protein sources that have a positive effect on the pup's health during their growth and development. In contrast, the higher level of ALT and AST has been linked with various health diseases such as hepatitis E, diabetes, chronic inflammation, cardiovascular disease, common bile duct stones, biliary obstructive diseases, chronic liver disease, stroke, risk of hepatocellular carcinoma, metabolic syndrome, hemochromatosis, anemic hypoxia, and malnutrition (Galvin et al., 2015; Martin & Friedman, 2018; Prati et al., 2002; Sherman, 1991).

**TABLE 3:** Effect of high egg protein on liver safety enzymes, kidney, and lipid profile in pups

Treatments	Liver safety enzymes (IU/L)		Kidney functions (IU/L)		Serum level (mg/dl)
	ALT	AST	Creatinine	BUN	Triglycerides
WF_0_	124.22 ± 1.56^a^	203.00 ± 1.11^a^	0.56 ± 0.07^a^	24.44 ± 1.33^a^	134.01 ± 1.73^d^
WF_1_	82.11 ± 1.27^c^	142.28 ± 1.22^b^	0.57 ± 0.08^a^	25.67 ± 2.00^a^	139.67 ± 1.80^c^
WF_2_	72.33 ± 1.58^b^	112.00 ± 1.39^c^	0.58 ± 0.05^a^	25.88 ± 2.36^a^	142.78 ± 1.71^b^
WF_3_	32.11 ± 0.78^d^	102.22 ± 1.98^d^	0.59 ± 0.05^a^	26.72 ± 2.15^a^	144.89 ± 1.26^a^

*Note*: WF_0_, control diet, 14% of soybean protein; WF_1_, 14% of egg protein; WF_2_, 16% of egg protein; and WF_3_, 18% of egg protein. ^A to D^Means with different superscripts differ significant effect (*p* ≤ .05).

Abbreviations: ALT, Alanine aminotransferase; AST, Aspartate aminotransferase; BUN, Blood urea nitrogen.

#### 3.1.3 Kidney functions

Kidney functions of pups were estimated through serum creatinine and blood urea nitrogen (BUN). These are the most common parameters used for measuring the impact of different quality protein levels on kidney functions. In this trial, all diets having a different level of egg protein (WF_1_, WF_2_, and WF_3_) with control plant source protein (WF_0_) showed a nonsignificant (*p* ≤ .05) effect on the kidney and their functions. However, the highest trend in the concentration of creatinine and BUN was observed in the pups with a high level of egg protein diet (WF_3_) (Table 3). The increased concentration of these kidney metabolites due to the normal range (0.4–0.8 mg/dl) did not affect the functioning of the kidney muscles (Baker et al., 1979; Salazar, 2014). It is concluded in some research that using whole egg and egg white did not affect kidney functions and its tissues (Chairuk et al., 2021; Saande et al., 2017).

#### 3.1.4 Serum triglyceride level

There was a significant difference (*p* ≤ 0.05) in serum triglycerides observed across all the treatments with different levels of egg and soybean proteins in comparison with control diet fed to the pups (Table 3). The highest serum triglycerides (*p* < 0.05) were observed in pups fed with a high level of egg protein (WF_3_). The trend showed that increased protein contents in the diet effect the level of serum triglycerides in pups. It was also observed that protein source, either it is a plant (134.01 ± 1.73 mg/dl) or animal (144.89 ± 1.26 mg/d) protein effects the formation of triglycerides metabolites. Our results corroborate those described by Khalighi Sikaroudi et al. (2020), who also observed a significant effect of egg consumption on serum triglyceride level in 57 clinical trials. In another research work by Lee et al. (2007), findings indicated that serum triglyceride level was elevated in rats fed with 5, 10, and 15% egg powder, which agrees with our results.

#### 3.1.5 Serum proteins

The main objective was to assess the protein levels in the serum in the pups for their standard growth and development. In this research, dietary increase in protein level could have a positive impact on pup's growth in terms of weight gain. Table 4 shows that all serum protein and its metabolites are in the normal range, which was in agreement with Giknis and Clifford (2008). However, increasing the level of dietary intake of egg protein effect the serum protein; the highest concentration of total protein, albumin, globulin, albumin/globulin, and transferrin was observed in the pups with the WF_3_ diet. Serum protein and metabolites were lowered in the pups fed the plant protein diet compared to animal source protein diet. The nonsignificant (*p* ≤ .05) results were observed in those pups who consumed the egg protein diets. The increasing trend of protein metabolites in the serum might be due to good protein contents in the egg (ovalbumin, ovoglobulins, ovotransferrin, and ovomucoid) compared to plant protein (Anton et al., 2009). Within animal sources, egg white protein was significantly higher than in the casein fed group (Matsuoka et al., 2017). The same results were observed by Gersovitz et al. (1980) and Lee et al. (2007) which showed that the level of egg protein also effect the concentration of serum albumin in young adults. Moreover, our results well agreed with the research work of Makrides et al. (2002) that the concentrations of transferrin in the egg yolk‐treated breastfed and formula‐fed infants (>37‐week gestation) were improved as compared to the control group within both the breastfed and formula‐fed cohorts. The improvements in serum proteins with egg treatment reflect an increase in iron, protein, and other vitamin transportation. Therefore, egg proteins are very effective against atopic disease, edematous malnutrition, inflammation, and weight loss in infants (Guha et al., 2019; Mine, 2015; Strixner & Kulozik, 2011).

**TABLE 4:** Effect of different levels of egg protein in weaning food of pups on blood protein

Treatments	Serum proteins (g/dl)				
	Total protein	Albumin	Globulin	Albumin/globulin	Transferrin
WF_0_	5.63 ± 0.14^c^	3.35 ± 0.12^b^	2.27 ± 0.23^b^	1.49 ± 0.20^a^	1.70 ± 0.01^d^
WF_1_	6.30 ± 0.15^b^	3.70 ± 0.14^a^	2.60 ± 0.21^a^	1.43 ± 0.17^a^	2.69 ± 0.04^c^
WF_2_	6.52 ± 0.13^a^	3.82 ± 0.09^a^	2.65 ± 0.23^a^	1.47 ± 0.22^a^	2.73 ± 0.03^b^
WF_3_	6.60 ± 0.08^a^	3.86 ± 0.18^a^	2.77 ± 0.15^a^	1.58 ± 0.10^a^	2.78 ± 0.02^a^

*Note*: WF_0_, control diet, 14% of soybean protein; WF_1_, 14% of egg protein; WF_2_, 16% of egg protein; and WF_3_, 18% of egg protein. ^A to D^Means with different superscripts differ significant effect (*p* ≤ .05).

#### 3.1.6 Immunity biomarkers

An increasing level of protein content in the diet of the pups effect the immunity biomarker that has been presented in Table 5. Immunity biomarkers (IgA, IgG, and IgM levels) were observed highest in the pups fed the WF_3_ diet. The maximum amount of IgA, IgG, and IgM was 0.055 ± 0.02, 76.33 ± 2.39, and 34.36 ± 1.02 mg/dl, respectively. Nonsignificant (*p* ≤ .05) result was observed in the immunity biomarkers fed WF_0_, WF_1_, and WF_2_ diets. There was a nonsignificant (*p* ≤ .05) difference in these immunity biomarkers of pups that might be due to the high biological value of soybean protein meal (74%) with comparison of egg protein. Moreover, the immunoglobulin E (IgE) level remained unchanged in all pups fed high levels of egg protein and plant protein diets. The type of immunoglobulin, especially IgA, has the potential to provide the first line of defense against pathogenic microbes, including viruses and bacteria (Awuchi, Akram, et al., 2022; Awuchi, Chukwu, et al., 2022; Woof & Kerr, 2006). The findings of current research work are similar to the study in which celiac disease patients were treated with egg protein powders for a period of 6 months. There was upregulation of jejunal IgA immunocytes in groups fed with different concentrations of egg protein substituted weaning foods (Dannaeus & Inganäs, 1981). The increased IgA level defend the body from allergy and atopic diseases. The serum IgG level was also increased with increasing the level of egg white in the diet of mice, and serum IgE levels remained unchanged in all mice groups (Song et al., 2014). According to the study by Burrows et al. (1989), the level of IgE in the blood serum rises which can be allergic if the blood serum IgE levels are low in the serum of blood which can cause many chronic infections. The increased level of IgG in those pups who were fed high egg protein contents having a positive impact in terms of their growth parameters like weight gain was higher because the high IgG level in blood and lymph fluid protects the body against bacteria and viruses and neutralizes bacterial toxins, triggers complement protein systems, and binds antigens to enhance the effectiveness of phagocytosis (Stapel et al., 2008).

**TABLE 5:** Effect of different levels of egg protein in weaning food of pups on immunity biomarkers

Treatments	Immunity biomarkers			
	IgA (mg/dl)	IgG (mg/dl)	IgM (mg/dl)	IgE (IU/ml)
WF_0_	0.038 ± 0.03^b^	57.88 ± 1.26^b^	33.05 ± 0.9^b^	<0.01 ± 0.10^a^
WF_1_	0.043 ± 0.04^b^	63.43 ± 1.35^b^	34.05 ± 0.72^b^	<0.01 ± 0.10^a^
WF_2_	0.047 ± 0.03^b^	68.15 ± 1.00^b^	34.11 ± 1.29^b^	<0.01 ± 0.10^a^
WF_3_	0.055 ± 0.02^a^	76.33 ± 2.39^a^	34.36 ± 1.02^a^	<0.01 ± 0.10^a^

*Note*: WF_0_, control diet, 14% of soybean protein; WF_1_, 14% of egg protein; WF_2_, 16% of egg protein; and WF_3_, 18% of egg protein. ^A to D^Means with different superscripts differ significant effect (*p* ≤ .05).

Abbreviations: IgA, Immunoglobulin A; IgG, Immunoglobulin G; IgM, Immunoglobulin M; IgE, Immunoglobulin E.

## 4 CONCLUSION

Evidence indicates that due to the addition of protein source in the infant's diet for making it balanced nutritional profile improve growth and development. The findings of the present research work demonstrate that the red and white blood cells were influenced by egg protein, whereas red and white blood cells were lower in the pups fed 14% of soybean protein compared to those fed 14%, 16%, and 18% of egg protein. The 18% of protein with the ratio of 75% egg protein and 25% soybean protein showed positive results on the blood metabolites for growth. In addition, increased egg protein levels in the diets affect the concentration of alanine aminotransferase and aspartate aminotransferase in pups. Ultimately, the successful clarification of the positive impact of egg protein in an infant's formula feed significantly improved the growth of weaning pups. Therefore, eggs have the potential to reduce the risk of protein energy malnutrition (PEM). Further studies are needed to study the impact of this formula feed on an infant's growth and development.

## FUNDING INFORMATION

The research work was funded by Pakistan Higher Education Commission (HEC) under Pakistan funded project (HEC/R&D/NRPU/2017/9287).

## CONFLICT OF INTEREST

All authors have no conflict of interest to declare.

## CONSENT FOR PUBLICATION

All authors agreed for publication of this manuscript.

## Data Availability

Even though adequate data have been given in the form of tables; however, all authors declare that if more data are required, then the data will be provided on a request basis.
